# Development of a System and Method for Automated Isolation of Stromal Vascular Fraction from Adipose Tissue Lipoaspirate

**DOI:** 10.1155/2015/109353

**Published:** 2015-06-08

**Authors:** Swathi SundarRaj, Abhijeet Deshmukh, Nancy Priya, Vidya S. Krishnan, Murali Cherat, Anish Sen Majumdar

**Affiliations:** Stempeutics Research Pvt. Ltd., Akshay Tech Park Nos. 72 and 73, 2nd Floor, EPIP Zone, Phase 1, Whitefield, Bangalore 560066, India

## Abstract

Autologous fat grafting for soft tissue reconstruction is challenged by unpredictable long-term graft survival. Fat derived stromal vascular fraction (SVF) is gaining popularity in tissue reconstruction as SVF-enriched fat grafts demonstrate improved engraftment. SVF also has potential in regenerative medicine for remodeling of ischemic tissues by promoting angiogenesis. Since SVF cells do not require culture expansion, attempts are being made to develop automated devices to isolate SVF at the point of care. We report development of a closed, automated system to process up to 500 mL lipoaspirate using cell size-dependent filtration technology. The yield of SVF obtained by automated tissue digestion and filtration (1.17 ± 0.5 × 10^5^ cells/gram) was equivalent to that obtained by manual isolation (1.15 ± 0.3 × 10^5^; *p* = 0.8), and the viability of the cells isolated by both methods was greater than 90%. Cell composition included CD34+CD31− adipose stromal cells, CD34+CD31+ endothelial progenitor cells, and CD34−CD31+ endothelial cells, and their relative percentages were equivalent to SVF isolated by the manual method. CFU-F capacity and expression of angiogenic factors were also comparable with the manual method, establishing proof-of-concept for fully automated SVF isolation, suitable for use in reconstructive surgeries and regenerative medicine applications.

## 1. Introduction

Adipose tissue represents a cell source with a distinct advantage for autologous therapies and stem cell banking. Fat tissue can be easily harvested by liposuction and the lipoaspirate, an otherwise discarded by-product of cosmetic surgery, can be processed by enzymatic or mechanical dissociation to obtain the component cellular fractions. The stromal vascular fraction (SVF) obtained by this process is a rich source of different types of stem and progenitor cells and has become central to an increasing array of applications in regenerative medicine [1]. SVF contains multipotent stromal cells called adipose-derived mesenchymal stromal cells (ASC), endothelial cells (EC), endothelial progenitor cells (EPC), pericytes, preadipocytes, and hematopoietic cells [2, 3]. This cocktail of progenitor cells in the SVF, particularly the ASC and the EPC polulations, is well documented to have angiogenic and neovasculogenic properties [4] which are being exploited in several clinical trials to bring about therapeutic angiogenesis [1]. The SVF also harbors mature cells such as fibroblasts, vascular smooth mucle cells, endothelial cells, lymphocytes, monocytes, red blood cells (RBC), and a small fraction of adipocytes [2, 3]. Many clinical trials have demonstrated safety [5–7]and efficacy of autologous SVF use in regenerative cell therapy for wound healing, skeletal regeneration, cardiovascular and peripheral vascular diseases, and tissue engineering [1, 8].

In addition, SVF has demonstrated significant potential in plastic and reconstructive surgery for soft tissue repair, reconstruction, and augmentation for therapeutic or aesthetic purposes [9, 10]. Clinical use of SVF in this field has developed at a particularly rapid pace over the past few years owing to the ease of access to subcutaneous adipose tissue for plastic surgeons. Common procedures where SVF has been used are postmastectomy breast reconstruction [11, 12], cosmetic breast augmentation [11, 13], facial restructuring [11, 14, 15], scar and deformity correction, and lipoatrophy treatment [16]. SVF-enriched fat grafting performed for such procedures has demonstrated improved engraftment of autologous fat grafts leading to several clinical practicioners adopting this as the preferred method for treating large volume soft- tissue defects. The activity of the ASC and EPC populations in the SVF has been postulated to bring about rapid angiogenesis and promote survival of the ischemic fat graft, while the preadipocyte population is believed to contribute to adipocyte turnover and maintain graft volume in the long run [17].

One of the main challenges for the entry of SVF based therapeutics to the clinic is the generation of a clinically acceptable grade of SVF cells with minimal manipulation. It is important to identify and control all possible factors that may affect the safety and quality of the SVF cell preparation, which should be performed in accordance with current good manufacturing practices (cGMP) guidelines. Isolation of SVF requires the fat to be harvested by liposuction and transportation to a cGMP-compliant laboratory for further processing. The various steps towards SVF isolation broadly include washing of the fat tissue to remove blood and tumescent fluids, enzymatic digestion, and centrifugation to recover the cells. Manual processing therefore requires expensive infrastructure and skilled technicians which are not available with most clinics. Many clinics procure SVF by transferring the fat to an external facility for cell isolation, which in turn entails storage, handling and transportation of fat and cells, and multiple patient visits. Consistency of handling of the tissue sample and safety of the bench process in an open system is also a concern. These challenges are being largely overcome by attempts to develop fully automated, point-of-care devices that can separate SVF from fat in a highly quality controlled and consistent manner.

In this report, we describe the design and development of an automated system for processing lipoaspirate to obtain SVF cells, intended for point-of-care use. Towards automation, we have developed a novel process for isolation of SVF by partitioning the SVF into the aqueous phase of the digest and subsequent recovery by membrane filtration. This process was first standardized by manual processing. A prototype device was then developed in accordance with the process requirements. The method was subsequently automated in the prototype device and the characteristics of the SVF obtained were determined. The SVF isolated using the system was validated for yield, viability, composition, clonal expansion capacity, and expression of angiogenic growth factors, in comparison against the manual process using centrifugation. The data presented here demonstrate that the SVF isolated by this automated process fulfils the expected criteria for potential therapeutic applications.

## 2. Materials and Methods

### 2.1. Human Adipose Tissue Samples

The study was conducted in accordance with the ethics committee and the committee for stem cell research and therapy of Manipal hospital, Bangalore, India (study number MIRM/002/08). Human adipose tissue samples were obtained with written informed consent from individuals undergoing elective cosmetic surgery at the department of plastic surgery, Manipal hospital. Lipoaspirate tissue was obtained from abdomen, thigh, or hip regions of both male and female donors (*n* = 11). Mean age of donors was 30.86 years (range 17–47 years), and average body mass index (BMI) 29.4 (range 26–35). All donors were of Indian ethnicity. Each tissue sample was simultaneously processed by both the manual and automated methods for all comparative studies of yield, viability, cell composition, and functional parameters.

### 2.2. Manual Isolation of SVF by Centrifugation of Whole Tissue Digest

SVF was isolated enzymatically from lipoaspirate tissue by digestion with collagenase [18]. Briefly, the aspirate was washed three or four times with lactated Ringer's solution and digested with collagenase NB-4 (SERVA Electrophoresis GmbH) in lactated Ringer's solution in standard tissue culture flasks (BD Falcon). Digestion was performed at 37°C with 5% humidified CO_2_ and continuous agitation for 60 min. The digest was then centrifuged for 20 min at 400 ×g. The supernatant containing adipocytes was discarded and the pellet containing the SVF was washed twice and filtered through a 100 *μ*m cell strainer (BD Falcon). The SVF cells were counted manually using a Neubauer chamber. Viability was determined by staining with 7-aminoactinomycin D (7-AAD) (BD Biosciences) and flow cytometry analysis (BD LSR II, BD Biosciences). Counting and viability analyses were performed in two replicates for each sample.

Verification of the accuracy of manual counting of SVF was performed by direct comparison of the manual counting method against counts obtained using an automated image based cell counter (Tali Image Cytometer, Life Technologies). Difference between the two methods was found to be not significant (see Supplementary Table 1 in Supplementary Material available online at http://dx.doi.org/10.1155/2015/109353).

### 2.3. Manual Isolation of SVF by Phase Separation and Filtration

Lipoaspirate tissue was washed three or four times with lactated Ringer's solution and digested with collagenase NB-4 (SERVA Electrophoresis GmbH) as above with continuous agitation. At the end of tissue digestion the digest was allowed to rest for a period of 10 min at room temperature by placing the tissue culture flasks in vertical position, to allow clear separation of the upper fatty and lower aqueous phases. The lower aqueous phase was then transferred to a fresh tube and centrifuged to obtain the SVF cell pellet in the initial experiments, to standardize the phase separation process. In subsequent experiments, the phase separation time was reduced to 2 min as the fatty and aqueous phases were found to partition efficiently within that time. To standardize the filtration process, the aqueous phase of the digest was serially passed through membrane filters of different porosity. Nylon filters (Millipore) of 100 *μ*m and 35 *μ*m pore size were used for prefiltration, and polycarbonate track etch (PCTE, Sterlitech corporation) filters of pore size 10 *μ*m, 8 *μ*m, 5 *μ*m, 3 *μ*m, or 2 *μ*m were used for final retention and recovery of SVF. The filter retentate was recovered and the SVF cells were counted manually using a Neubauer chamber. Viability of SVF in the retentate was determined by staining with 7-AAD (BD Biosciences) and flow cytometry analysis (BD LSR II, BD Biosciences). Counting and viability analysis was performed in two replicates for each sample. Composition of the SVF was determined by immunophenotyping and flow cytometry analysis.

### 2.4. Automated Isolation of SVF

The main operational modules of the automated system (patent pending) comprised of a tissue digestion chamber, heating and agitation mechanism, and a three-stage filtration system. The tissue digestion chamber was designed to provide a maximum surface area to volume ratio to enable maximal contact between the fat and enzyme layers. Architectural elements in the chamber along with an orbital agitation mechanism were designed to enable efficient mixing of the fat and the enzyme. The agitation mechanism was equipped to lift the digestion chamber to a vertical position to allow phase separation. The geometry of the digestion chamber was also ideal to provide maximal height for the efficient partitioning, separation, and drainage of the aqueous and fatty phases of the contents as required. The filtration system comprised of multiple filtration units, each with a filtration capacity of 100 mL. A single filtration unit comprised of a serial arrangement of three filter membranes of 100 *μ*m, 35 *μ*m, and 5 *μ*m porosity. The filtration unit was fitted with a vibration mechanism to facilitate filtration. The entire system was programmed to be controlled through a digital user interface. Flow of tissue and liquids was controlled using peristaltic pumps and pinch valves.

The lipoaspirate tissue samples were transferred into the tissue digestion chamber, and the various steps of washing, digestion, and filtration were carried out in an automated fashion. The collected SVF was recovered from the filter chambers using a syringe. The SVF cells were counted manually using a Neubauer chamber. Viability of SVF was determined by staining with 7-AAD (BD Biosciences) and flow cytometry analysis (BD LSR II, BD Biosciences). Counting and viability analysis was performed in two replicates for each sample. Composition of the SVF was determined by immunophenotyping and flow cytometry analysis.

### 2.5. Immunophenotyping

The following fluorochrome conjugated antibodies were used to label cell surface antigens: CD34 PE-Cy7, CD31 APC, CD73 PE, CD146 PE, CD45 PE, HLA DR FITC, and glycophorin A PE (BD Pharmingen). The relevant mouse isotypes (BD Pharmingen) were used as control. Cells were stained with labelled antibodies in the dark at 4°C for 45 min. A minimum of 30 000 events were acquired on a BD LSR II flow cytometer and the results were analyzed using BD FACSDiva software.

### 2.6. CFU-F Assay

Freshly isolated SVF cells were seeded in Dulbecco's modified Eagle's medium (DMEM; Invitrogen) supplemented with 10% fetal bovine serum (FBS; Hyclone), 2 mM glutamine, and antibiotics (Invitrogen) in six-well tissue culture plates (BD Falcon) at plating densities of 2000, 1000, and 500 cells/cm^2^. The SVF were plated in duplicates for each plating density. After 9 days of incubation, the cells were washed with PBS, fixed in 1% paraformaldehyde for 20 min, stained with 0.1% toluidine blue (in 1% formaldehyde solution) for 1 h on a shaker, and then rinsed with water. The numbers of colony-forming unit-fibroblasts (CFU-F) were then counted where aggregates of 50 cells or more were defined as CFU-Fs.

### 2.7. RT-PCR Validation

Total RNA was extracted using an RNeasy mini kit (Qiagen), treated with DNA free DNase I (Ambion), reverse transcribed using a Superscript III first strand kit (Invitrogen), and the cDNA used for PCR. 18s ribosomal RNA expression was used to normalize the cDNA concentrations for all sample sets. The primer sequences used and amplicon size are listed in Table 1. Gene expression analysis was carried out in duplicate sets for each sample.

### 2.8. Statistical Analyses

The differences between corresponding pairs of data sets for SVF isolated by manual and automated methods were examined for statistical significance using the paired Student's *t*-test; *p* ≤ 0.05 was considered significant.

## 3. Results

Freshly obtained lipoaspirate tissues were divided into two halves for further processing. As described in the Materials and Methods section, one half of the tissue was manually washed and digested following which the separated aqueous phase of the digest was centrifuged to obtain the SVF. In parallel, the other half of the tissue was processed by the conventional manual method wherein the whole tissue digest comprising fatty and aqueous components was centrifuged to obtain the SVF cell pellet.

### 3.1. Recovery of SVF into Aqueous Phase of Digest and Scale-Up

No statistically significant differences were observed between the yields of SVF obtained from the separated aqueous phase alone, as compared to centrifugation of the whole tissue digest (Table 2). Recovery of SVF from the aqueous phase was tested to be efficient for volumes ranging from 50 to 500 mL of tissue processed. The mean SVF yield obtained from the aqueous phase of the digest ranged from 0.89 to 2.00 × 10^5^ cells per gram of adipose tissue for all volumes of fat processed, and this yield ranges between 87 and 117% of the concomitant yield obtained by centrifugation of the whole tissue digest. The SVF obtained by both the methods was also evaluated for the number of colony forming progenitors by CFU-F assay (Table 3). The number of CFU-F derived from the SVF isolated from the aqueous phase alone (mean CFU-F per 100 cells = 0.45) was found to be comparable with that obtained by centrifugation of the whole tissue digest (mean CFU-F per 100 cells = 0.41) (Table 3). The results clearly show that the clonal expansion capacity in the SVF isolated using the two methods was equivalent. Therefore, recovery of SVF by sedimentation into the aqueous phase of the digest was determined to be an efficient method for further automation and was scalable from a volume of 50 mL to 500 mL lipoaspirate.

### 3.2. Recovery of SVF by Retention on Membrane Filters

**Figure 1:**
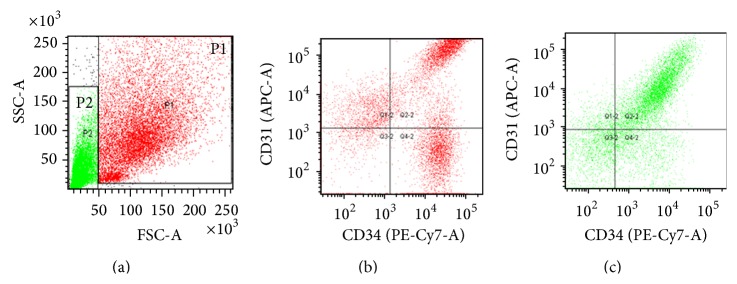
Detection of ASC, EPC, and EC populations in SVF with respect to cell size. The figure shows a representative flow cytometry scatter profile for SVF cells that have been stained with fluorescent tagged antibodies against the cell surface markers CD31 and CD34. (a) Forward scatter (FSC) and side scatter (SSC). Events in gates P1 and P2 represent the FSC^hi^ and FSC^lo^ populations, respectively. (b) Detection of FSC^hi^ cells expressing CD31 and CD34. (c) Detection of FSC^lo^ cells expressing CD31 and CD34.

Relative differences in cell size between the different cell types in the SVF was detected by measuring their light scattering properties by flow cytometry. The major cell types known to be present in the SVF were further distinguished by their surface marker profile as follows: ASC (CD31^−^CD34^+^), EPC (CD31^+^CD34^+^), and EC (CD31^+^CD34^−^). From the light scatter profile (Figure 1) the ASC, EPC, and EC populations were found to have high forward scatter (FSC^hi^) and could be distinguished from the debris which showed low forward and side scatter properties (FSC^lo^ and SSC^lo^). Some EPC were also found in the FSC^lo^ region indicating that the EPC are smaller than the ASC and EC. These results suggest that all the regenerative cell populations in the SVF, namely, the ASC, EPC, and EC, could be separated from cellular debris by retention on a membrane filter.

**Figure 2:**
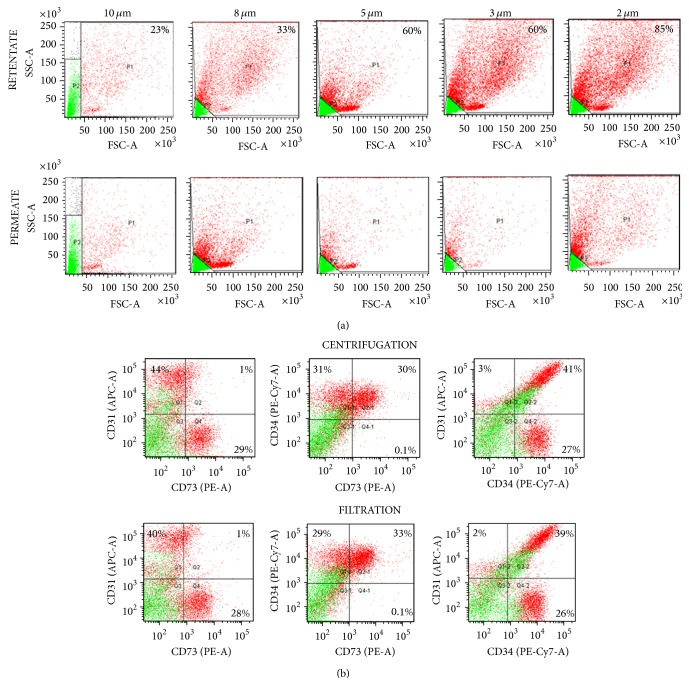
SVF recovery by filtration. (a) The figure shows a representative flow cytometry light scatter profile for SVF cells that have been recovered from the retentate and permeate fractions of filters of indicated pore size in *μ*m. Percentage values represent the proportion of cells retained on the filter membrane relative to the total cell number in the whole SVF before filtration. Data shows a representative set of light scatter plots from four individual donors in each group. Events in gates P1 (red) and P2 (green) represent the FSC^hi^ and FSC^lo^ populations, respectively. (b) Antibody labelling and flow cytometric detection of cell surface markers expressed by SVF cells recovered by centrifugation or filtration using 5 *μ*m PCTE filter. The percentage positive cells for each marker combination is calculated after subtraction of the nonspecific fluorescence obtained with the isotype controls. Data shows a representative set of plots from four individual donors in each group. Plots represent events from both FSC^hi^ (red) and FSC^lo^ (green) populations.

In order to recover SVF cells without centrifugation, the separated aqueous phase of the digest was passed through filters of various biocompatible material and porosity to select the filter specifications for retention of the desired cell populations from the SVF. The aqueous fraction was first prefiltered sequentially through woven nylon filters of 100 *μ*m and 35 *μ*m pore size to remove coarse material such as undigested tissue, collagen fibers, and cell aggregates. The filtrate was then loaded onto polycarbonate track etch (PCTE) filters of different pore sizes ranging from 10 *μ*m to 2 *μ*m. The fractionation of the different SVF cell components between the retentate (collected on the filter) and the permeate (flow-through) was then detected by flow cytometry. Greater than 60% of the SVF cells were recovered in the retentate on PCTE filters of pore size 5 *μ*m and below (Figure 2(a)), while considerable loss of cells was observed where 10 *μ*m and 8 *μ*m filters were used. A specification of 5 *μ*m was therefore selected as the optimal pore size for maximal recovery of SVF cells by filtration from the aqueous phase of the tissue digest. Retention of cells on the 5 *μ*m filter was visualized by staining with Toluidene blue. Representative images for the stained filters is provided in Supplementary Figure 1.

The composition of SVF recovered by aqueous phase filtration through the 5 *μ*m PCTE membranes was compared against SVF recovered by centrifugation of the whole tissue digest and the results were found to be highly similar (Figure 2(b)). The average proportions of the different SVF components from centrifugation and filtration methods was determined to be 22 ± 7% and 26 ± 3%, respectively, for CD31^−^CD34^+^ ASC, 36 ± 10% and 31 ± 10%, respectively, for CD31^+^CD34^+^ EPC, and 3 ± 1% and 3 ± 2%, respectively, for CD31^+^CD34^−^ EC populations. These results led us to conclude that separation of the aqueous phase containing SVF from the fat fraction, followed by recovery of the cells on membrane filters is feasible and warrants further development towards automation.

### 3.3. Process Automation

**Figure 3:**
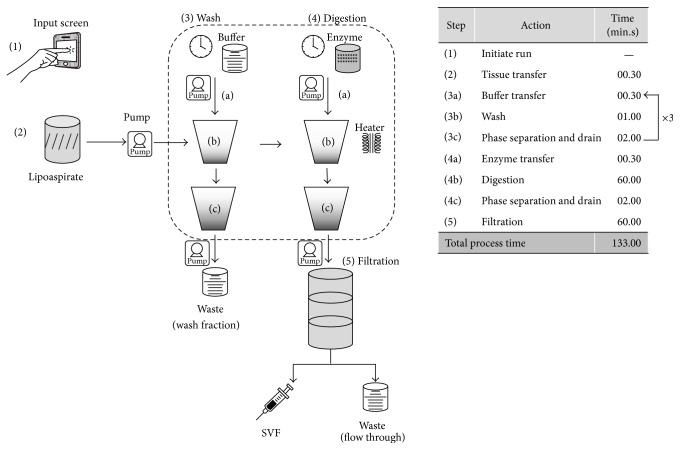
Schematic representation of the automated process for isolation of SVF from lipoaspirate tissue. The schematic represents the various steps involved in SVF isolation. (1) The automated run is initiated by the user via a graphical user interphase; (2) lipoaspirate tissue is transferred into the tissue processing unit with the help of pump; (3) wash sequence comprises of (3a) transfer of buffer into the tissue processing unit with the help of pump, (3b) washing of tissue by agitation, and (3c) phase separation into upper fat and lower aqueous phases, followed by removal of the lower aqueous phase. The wash sequence is repeated thrice for complete removal of blood and tumescent fluids from the fat tissue; (4) the tissue is enzymatically digested. A digestion sequence comprises of (4a) transfer of enzyme into the tissue processing unit with the help of pump, (4b) the tissue is digested under controlled temperature and constant agitation, (4c) phase separation into upper fat and lower aqueous phases, and (5) the aqueous fraction of the digest is concentrated by filtration to deliver the SVF. The time taken for each of the process steps is shown in the accompanying table. Total process time is 133 min.

A tissue digestion chamber, heating and agitation mechanism, and multistage filtration units were developed towards automation of the various steps involved in SVF isolation, namely (Figure 3), washing and digestion of the lipoaspirate tissue, aqueous phase separation, cell concentration by sequential filtration through nylon filters of 100 *μ*m and 35 *μ*m pore size, and ultimate recovery of the SVF on 5 *μ*m PCTE filters. The device was programmed to process 500 mL of lipoaspirate tissue. Effect of peristaltic pump was initially studied to ensure that the pump action does not cause mechanical damage to the tissue and cells. The yield and viability of SVF isolated from the lipoaspirate tissue before and after passing through the peristaltic pump was found to be comparable (Supplementary Table 2, *p* = 0.99 by paired *t*-test, *n* = 3).

**Figure 4:**
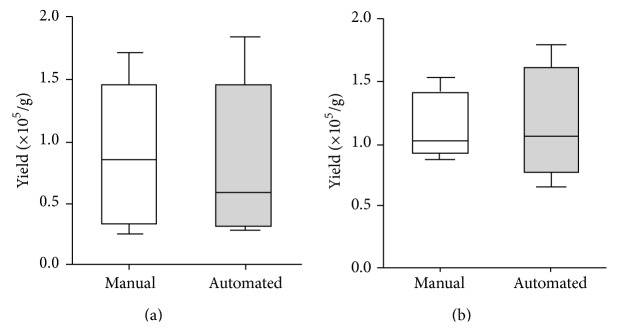
Comparison of SVF yield from manual and automated process. Yield of SVF obtained by automated digestion (a) and filtration (b) as compared to the manual process of cell isolation using centrifugation technology. Data is represented as mean ± SD for four individual samples tested. Difference in cell yield between manual centrifugation method and automated filtration is not significant (*p* = 0.8, paired *t*-test).

**Figure 5:**
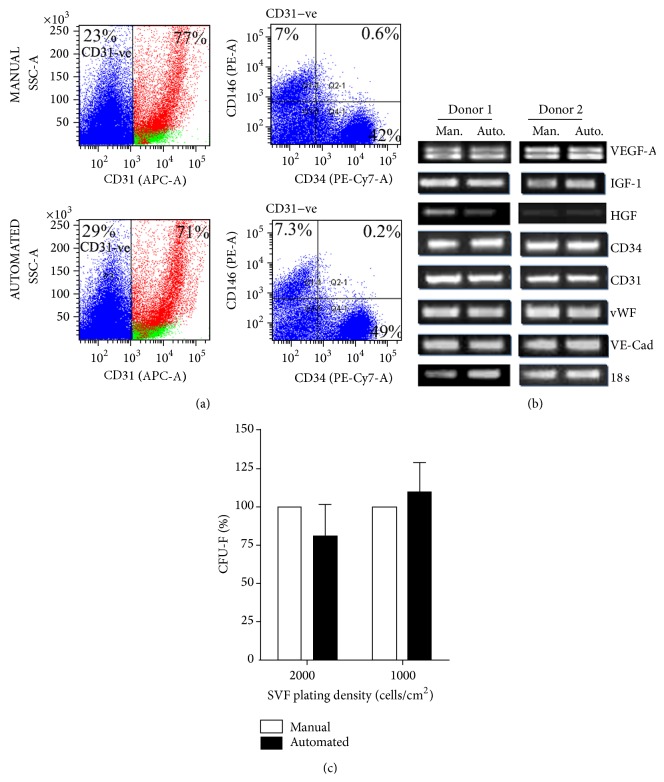
Phenotypic and functional characterization of SVF isolated by the manual and automated process. (a) Detection of pericytes in SVF isolated by the manual and automated process. The CD31−ve cells in the SVF were gated (depicted in blue) and analyzed for expression of CD34 and CD146. Values represent percentage of CD31−ve cells expressing CD34 or CD146. Data shows a representative set of plots from four individual donors in each group. (b) Semiquantitative RT–PCR analysis for expression of angiogenic markers in SVF obtained from manual and automated process. 18s ribosomal RNA expression was used to normalize cDNA concentration for each sample set. Data depicts gene expression in two pairs of samples out of four pairs of individual donors analysed. Man: manual; Auto: automated. (c) Clonal expansion potential of SVF obtained by manual and automated process. Data represents mean number of CFU-F obtained for each plating density from four individual donors, each assayed in duplicate. Errors represent SD from four individual donors.

Individual subsystems developed for tissue digestion and filtration were tested independently to determine the efficiency of the automated process in comparion with manual SVF isolation by centrifugation. The time taken for each of the process steps is shown in the accompanying table in Figure 3. The total process time from initiation of the run to recovery of the SVF was found to be 133 min. The mean volume of SVF recovered from a single filter unit was found to be 10.8 mL (range 4–20 mL). The yield and viability of the SVF cells obtained by automated tissue digestion and filtration using the device was found to be equivalent (*p* = 0.8, *n* = 5, paired *t*-test) to that obtained by the manual process (Figures 4(a) and 4(b); Table 4). The average yield from the automated isolation was found to be 94 ± 28% for the digestion and 98 ± 21% for the filtration systems, respectively, when compared to the baseline (100%) yields obtained with the manual process; the viability of the cells isolated by both methods was consistently greater than 90%. The composition of the SVF cells recovered from the automated process was determined by flow cytometric analysis and was found to be comparable with the conventional manual process (Table 5). In particular, the mean percentage of CD31^−^CD34^+^ cells from the automated process was 25 ± 9% as compared to 20 ± 5% from the manual process, and the percentage of CD31^+^CD34^+^ cells obtained was 24 ± 6% and 30 ± 10%, respectively, for the automated and manual methods. Adipose tissue is also reported to contain a population of pericytes that are CD31^−^CD146^+^ [19]. For detection of pericytes, the CD31−ve cells in the SVF were gated (Figure 5(a), upper and lower left panels) and analyzed for the expression of CD34 and CD146 (Figure 5(a), upper and lower right panels). As shown in Figure 5(a), the percentage of CD34−CD146+ cells within the CD31−ve population of the SVF was found to be comparable between cells isolated by the manual and automated methods. The percentage of CD31−CD146+ cells in the total SVF was also found to be equivalent between the manual process (1.9 ± 1.2%) and the automated process (2.0 ± 1.4%). These observations confirm that the SVF isolated using the automated device contains the relevant proportions of ASC, EPC, and pericyte populations.

Gene expression analysis by semiquantitative RT-PCR revealed that the expression levels of vascular endothelial growth factor (VEGF-A), hepatocyte growth factor (HGF), insulin-like growth factor-1 (IGF-1), CD31, CD34, von Willebrand factor (vWF), and vascular endothelial cadherin (VE-Cadherin) in the SVF cells isolated by the automated process were comparable with the manually isolated SVF (Figure 5(b)). Although minor differences in the expression levels of certain genes, for example, HGF, were observed between different donors, there were no remarkable differences between SVF isolated using manual or automated methods. The number of CFU-F derived from SVF isolated by the automated system was also found to be comparable with the SVF obtained by manual processing (Figure 5(c)).

Taken together, these data suggest that the yield and viability of the SVF cells, as well as their phenotypic composition, CFU-F potential and angiogenic gene expression profile are equivalent between the manual and automated processes.

## 4. Discussion

SVF harbors enormous therapeutic potential for a multitude of regenerative and reconstructive applications. However, its use can be limited by the numerous challenges associated with obtaining clinical grade SVF cells. These include lack of c-GMP compliant infrastructure, physician know-how and skilled technical staff, time taken for isolation, and logistic hurdles, all of which can impact the sterility, viability, functionality, and consistency of the cell preparation. These challenges can be overcome by the availability of an automated, closed system for cell isolation that can be used at the clinic to deliver SVF cells in a highly quality controlled and consistent manner.

Following enzymatic digestion of the fat tissue, centrifugation of the whole digest is the conventional and most widely used process for recovery of SVF, both by manual processing [18] and in existing automated systems [11, 20]. We aimed at developing a system and process for isolating clinical grade SVF cells without the use of a centrifuge in order to decrease the device footprint and develop a gentle cell isolation process. Towards this aim, we have developed a novel process for SVF isolation wherein the aqueous phase of digested adipose tissue was separated from the digested fat and the SVF cells were recovered from the aqueous phase by retention on a membrane filter. Device development was then initiated by design and prototyping of the main operational modules comprising the tissue digestion chamber, heating and agitation mechanism, and a three-stage filtration unit. The entire system was programmed to be controlled through a digital user interface. The system was then used to digest and recover SVF from input lipoaspirate tissue. The SVF isolated using the system was validated for yield, viability, composition, clonal expansion capacity, and expression of angiogenic growth factors, in comparison with the manual process using centrifugation.

The partitioning of the SVF into the aqueous phase of the tissue digest was found to be efficient, as the yield, viability, cell composition, and CFU-F capacity of SVF recovered by such fractionation was observed to be equivalent to the conventional centrifugation method. The yield of SVF per gram of adipose tissue from our process (1-2 × 10^5^) also compares well with those reported for the Celution (2–2.5 × 10^5^) and Multistation (1 × 10^5^) systems [20] and is far superior to yields reported for the Cha-station (<2.5 × 10^4^) and Lipokit (<5 × 10^4^) [20], all of which are systems developed for point-of-care isolation of SVF.

Lipofilling procedures for soft-tissue reconstruction can require a range of volumes of fat tissue for grafting depending on the volume of the defect or the desired extent of augmentation. While facial lipoatrophy can be addressed by grafting as low as 10–20 mL of fat tissue, breast augmentation procedures might require up to 200–300 mL of the fat graft for a single breast [11–13]. Although the ratio of SVF to be supplemented to a given volume of fat graft has still not been systematically studied in humans, supplementation of SVF recovered from a corresponding volume of fat tissue has been widely reported to be safe and provides favorable clinical outcomes [11–16]. Currently available automated systems for SVF isolation are limited by the volume of fat that can be used. The maximal processing capacity for the fully automated Celution [20] and Tissue Genesis Cell Isolation Systems [11] have been reported to be 360 mL and 60 mL fat tissue, respectively. Although the Multistation has a higher capacity of 400 mL fat, it is a manually operated system. The mean processing time reported for Celution, Multistation, Cha-station, and Lipokit ranges from 88 to 115 minutes [20], while the Tissue Genesis Cell Isolation System has been reported to have a shorter processing time of 65 min; its processing capacity is also considerably lower [11]. Based on the literature cited above, the device reported in this study can process a larger volume of fat than other isolation systems within a reasonable time frame of 133 min. These factors, combined with the advantage of complete automation, make the device extremely viable for use in the operating room and potentially address a wide range of clinical applications.

Selection of the filter specifications was optimized to obtain an SVF yield, viability, and composition equivalent to that obtained using the conventional centrifugation method. A three-stage continuous filtration system fitted with a mechanical agitation unit was developed, wherein the aqueous phase is prefiltered through 100 *μ*m and 35 *μ*m nylon filters and the SVF is ultimately recovered on a 5 *μ*m PCTE filter. Undigested tissue, cell and tissue aggregates, and collagen fibers are separated from the SVF during 1st and 2nd stage filtration. Our results demonstrate that the cellular debris is also separated from the SVF which is retained on the 3rd filter. Centrifugation, on the other hand, would result in sedimentation of all dense material including aggregates and fibrous tissue along with the SVF cells. Therefore, we conclude that the filtration system employed has an advantage over centrifugation-based methods in obtaining SVF cells that are free from tissue aggregates and collagen fibers, which could pose complications in injection of SVF during surgery.

Composition of SVF obtained by the automated process was found to include CD34+CD31− ASC, CD34+CD31+ endothelial progenitor cells, CD34−CD31+ mature endothelial cells, and CD31−CD146+ pericytic cells. The relative percentages of the different cell types were comparable to SVF isolated by the manual centrifugation-based method. The functionality of the SVF isolated by the automated process was demonstrated by the ability to form colony forming units (CFU-F) representing self-renewal capacity of the ASC contained in the SVF. Gene expression analysis confirmed the presence of endothelial and progenitor cells from the expression of CD31, CD34, VE-cadherin, and von Willebrand factor. Production of angiogenic and antiapoptotic growth factors was also confirmed from expression of VEGF and IGF in the SVF.

## 5. Conclusions

In conclusion, we have successfully demonstrated proof-of-concept for automated isolation of SVF using a prototype device and a xeno-free isolation process. We are currently working towards development of a beta unit that will be validated for efficacy, sterility, and safety requirements prior to pilot human trials. Concentration of clinical grade SVF cells without the use of a centrifuge would deliver a compact system with a small footprint that can be easily accommodated in a clinical setting. In a country like India and other developing countries where lack of infrastructure and physician awareness pose huge hurdles for the adoption and penetration of SVF based interventions in the clinic, such a point-of-care device can provide considerable benefit and access to SVF.

## Supplementary Material

Supplementary Table 1: Verification of the accuracy of manual counting of SVF was performed by direct comparison of the manual counting method against counts obtained with an automated image based cell counter. Difference between the two methods is not significant (*p*=0.19, paired t-test).
Supplementary Table 2: Effect of peristaltic pump was studied to ensure that the pump action does not cause mechanical damage to the tissue and cells. The yield and viability of SVF isolated from the lipoaspirate tissue before and after passing through the peristaltic pump was found to be comparable (Supplementary table 2).
Supplementary Figure 1: Retention of cells on the 5 μm filter was visualized by staining with Toluidene blue. Representative images for the stained filters is provided in Supplementary figure 1.

## Figures and Tables

**Table 1:** Primer sequence used for semiquantitative RT-PCR analysis.

Gene	Forward primer	Reverse primer	Ta	Size
			°C	(bp)
18S	5′-CGGCTACCACATCCAAGGAA-3′	5′-GCTGGAATTACCGCGGCT-3′	56	186
CD31	5′-CAGGGTGACACTGGACAAGA-3′	5′-GGAGCAGGACAGGTTCAGTC-3′	59	650
CD34	5′-AATGAGGCCACAACAAACATCACA-3′	5′CTGTCCTTCTTAACCTCCGCACAGC-3′	57	400
VEGF-A	5′-CGAAGTGGTGAAGTTCATGGATG-3′	5′TTCTGTATCAGTCTTTCCTGGTGAG-3′	60	476
IGF-1	5′-GACATGCCCAAGACCCAGAAGGA-3′	5′-CGGTGGCATGTCACTCTTCACTC-3′	63	118
HGF	5′-ATGCATCCAAGGTCAAGGAG-3′	5′-TTCCATGTTCTTGTCCCACA-3′	61	349
v-WF	5′-TAAGTCTGAAGTAGAGGTGG-3′	5′-AGAGCAGCAGGAGCACTGGT-3′	59	100
VE-Cad	5′-GTGGAAGCGCGAGATGCCCA-3′	5′-AGCGTCCTGGTAGTCGCCCC-3′	59	237

Ta: annealing temperature; bp: base pair.

**Table 2:** SVF yield from lipoaspirates processed by whole digest centrifugation and aqueous phase separation.

Volume of fat	Centrifugation yield	Phase separation yield
(mL)	(×10^5^/gram)	(×10^5^/gram)
50 mL	2.05 ± 0.7 **(100%)**	2.00 ± 0.4 **(101 ± 17%)**
100 mL	1.45 ± 0.8 **(100%)**	1.48 ± 0.6 **(99 ± 08%)**
200 mL	1.00 ± 0.2 **(100%)**	0.89 ± 0.1 **(87 ± 27%)**
500 mL	1.32 ± 0.6 **(100%)**	1.6 ± 1.0 **(117 ± 18%)**

Cell yield is represented as mean cell number per gram of adipose tissue from three to seven donor samples processed for each volume. Percentage values indicate percentage recovery by aqueous phase separation as relative to centrifugation of the whole tissue digest. Data is represented as mean ± SD.

**Table 3:** Fibroblastic colony forming unit (CFU-F) assay for SVF recovered by aqueous phase separation as compared to whole digest centrifugation.

Plating density	Centrifugation	Phase separation
(SVF cells/10 cm^2^)		
20000	72 ± 16	101 ± 24
10000	48 ± 14	42 ± 16
5000	20 ± 6	21 ± 5

Data represents mean number of CFU-F obtained for each plating density from three individual donors, each assayed in duplicate. Errors represent SD from three individual donor samples.

**Table 4:** Yield and viability of SVF obtained by the automated process using filtration technology as compared to the manual process of cell isolation using centrifugation.

	Digestion		Filtration	
	Manual	Automated	Manual	Automated
SVF yield (×10^5^/gram)	0.90 ± 0.5	0.82 ± 0.6	1.15 ± 0.3	1.17 ± 0.5
Percentage yield	100%	94 ± 28%	100%	98 ± 21%
Percentage viability	96 ± 3.0%	96 ± 2.1%	97.3 ± 1.5%	97.5 ± 2.8%

Data is represented as mean ± SD for five individual samples tested. SVF Yield is represented as cell number ×10^5^/gram of adipose tissue. Percentage yield indicates percentage recovery by automated processing relative to the yield from manual processing taken as the baseline comparator. Difference in cell yield between manual centrifugation method and automated filtration is not significant (*p* = 0.8, paired *t*-test).

**Table 5:** Composition of SVF isolated by the automated process as compared to manual isolation.

Cell type	Marker profile	Manual	Automated
ASC	CD34^+^CD31^−^	20 ± 5	25 ± 9
EPC	CD34^+^CD31^+^	30 ± 10	24 ± 6
Mature EC	CD34^−^CD31^+^	4 ± 3	5 ± 1
Pericyte	CD31^−^CD146^+^	1.9 ± 1.2	2.0 ± 1.4
Lymphocytes	HLA DR^+^	24 ± 10	27 ± 10
RBC	GpA^+^	12 ± 5	15 ± 11

Table represents mean percentage positive cells with standard error, from four different data sets. ASC: adipose derived stem/stromal cells; EPC: endothelial progenitor cells; RBC: red blood cells.

## Abbreviations

7-AAD: 7-Aminoactinomycin D
ASC: Adipose derived stem/stromal cell
BMI: Body mass index
CFU-F: Colony-forming unit-fibroblasts
cGMP: Current good manufacturing practices
DMEM: Dulbecco's modified Eagle's medium
EC: Endothelial cell
EPC: Endothelial progenitor cell
FBS: Fetal bovine serum
FSC: Forward scatter
HGF: Hepatocyte growth factor
IGF: Insulin like growth factor
PCTE: Polycarbonate track etch
RBC: Red blood cell
RT-PCR: Reverse transcriptase polymerase chain reaction
SSC: Side scatter
SVF: Stromal vascular fraction
VE-cadherin: Vascular endothelial cadherin
VEGF: Vascular endothelial growth factor
vWF: von Willebrand factor.
