# Supplementary material for: Development of a System and Method for Automated Isolation of Stromal Vascular Fraction from Adipose Tissue Lipoaspirate
Source: Stem Cells Int. 2015 Jun 8;2015:109353. doi: 10.1155/2015/109353 (PMC4475713; doi:10.1155/2015/109353)
Supplement: Supplementary file 1 — Supplementary Table 1: Verification of the accuracy of manual counting of SVF was performed by direct comparison of the manual counting method against counts obtained with an automated image based cell counter. Difference between the two methods is not significant (p=0.19, paired t-test). Supplementary Table 2: Effect of peristaltic pump was studied to ensure that the pump action does not cause mechanical damage to the tissue and cells. The yield and viability of SVF isolated from the lipoaspirate tissue before and after passing through the peristaltic pump was found to be comparable (Supplementary table 2). Supplementary Figure 1: Retention of cells on the 5 μm filter was visualized by staining with Toluidene blue. Representative images for the stained filters is provided in Supplementary figure 1. [file 109353.f1.pdf]

Supplementary Table 1: Comparison of SVF cell counts obtained by manual counting vs. automated counting methods.

Sample ID	Manual count ($x 10^6$)	Automated count ($x 10^6$)
**Sample 1**	2.56	2.09
**Sample 2**	0.52	0.69
**Sample 3**	1.81	1.93
**Sample 4**	1.89	1.71
**Sample 5**	2.77	1.64
**Sample 6**	8.05	3.43
**Sample 7**	5.55	6.14
**Sample 8**	6.45	5.17
**Mean±SD**	**3.7±2.6**	**2.8±1.9**

Supplementary Table 2: Yield and viability of SVF from lipoaspirates processed before and after passing through peristaltic pump.

Sample ID	Control		Per. Pump.	
	Yield ($x10^5$ /gram)	Viability %	Yield ($x10^5$ /gram)	Viability %
**Sample 1**	2.4	95%	1.7	94%
**Sample 2**	2.6	98%	3.0	98%
**Sample 3**	0.56	98%	0.87	98%
**Mean±SD**	**1.85±1.1**	**96.7±2.3%**	**1.86±1.1**	**97.0±1.7%**

Difference between the control and per. pump. groups is not significant (p=0.99, paired t-test).

Supplementary figure 1

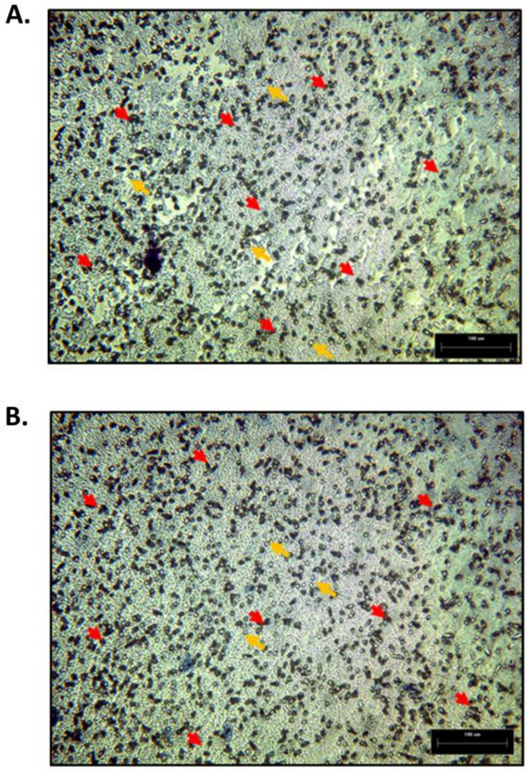

Supplementary figure 1. Retention of SVF cells on 5 um polycarbonate track etch filters (PCTE). The aqueous phase of digested lipoaspirate tissue was sequentially filtered through nylon filters of 100 µm and 35 µm pore size and SVF cells were ultimately recovered on a 5 µm PCTE filter. The 5 µm PCTE filter was then fixed in 1% paraformaldehyde for 20 min, stained with 0.1% toluidine blue (in 1% formaldehyde solution) for 1 h and then rinsed with water. Cells retained on the filter were visualized by light microscopy. Micrographs show a representative set of images from two different samples (A) & (B). Arrowheads indicate stained SVF cells (red) and filter pores (yellow). Scale bars=100 µm.
